# MALVA: Genotyping by Mapping-free ALlele Detection of Known VAriants

**DOI:** 10.1016/j.isci.2019.07.011

**Published:** 2019-07-12

**Authors:** Luca Denti, Marco Previtali, Giulia Bernardini, Alexander Schönhuth, Paola Bonizzoni

**Affiliations:** 1 Department of Computer Sciences, Systems and Communications, University of Milan-Bicocca, Piazza dell’Ateneo Nuovo, 1, 20126 Milan, Italy; 2 Centrum Wiskunde & Informatica, Science Park 123, 1098 XG Amsterdam, The Netherlands

**Keywords:** Biological Sciences, Genetics, Genomics, Bioinformatics

## Abstract

The amount of genetic variation discovered in human populations is growing rapidly leading to challenging computational tasks, such as variant calling. Standard methods for addressing this problem include read mapping, a computationally expensive procedure; thus, mapping-free tools have been proposed in recent years. These tools focus on isolated, biallelic SNPs, providing limited support for multi-allelic SNPs and short insertions and deletions of nucleotides (indels). Here we introduce MALVA, a mapping-free method to genotype an individual from a sample of reads. MALVA is the first mapping-free tool able to genotype multi-allelic SNPs and indels, even in high-density genomic regions, and to effectively handle a huge number of variants. MALVA requires one order of magnitude less time to genotype a donor than alignment-based pipelines, providing similar accuracy. Remarkably, on indels, MALVA provides even better results than the most widely adopted variant discovery tools.

## Highlights

• MALVA is able to genotype multi-allelic SNPs and indels without mapping reads
• MALVA calls correctly more indels than the most widely adopted genotyping pipelines
• Mapping-free approaches are as accurate as alignment-based ones, while being faster

## Graphical Abstract

**Figure:**
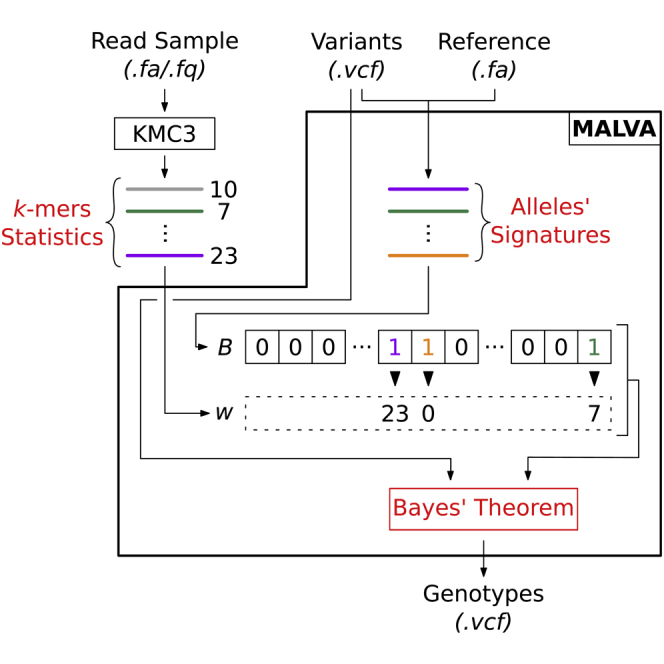

## Introduction

The discovery and characterization of sequence variations in human populations is crucial in genetic studies. A prime challenge is to efficiently analyze the variations of a freshly sequenced individual with respect to a reference genome and the available genomic variations data. To reach this goal, the standard pipeline includes aligning sequenced reads with software like BWA (Li and Durbin, 2009) and Bowtie (Langmead and Salzberg, 2012) and then calling the genotypes (e.g., with GATK [McKenna et al., 2010] or BCFtools [Li, 2011]); such an approach, though, can be highly time consuming and is thus impractical for clinical applications, where time is often an issue. Typically, in diploid organisms variant calling requires SNPs and indel detection and the identification of the pairs of alleles for each position of the studied genome, called genotype.

Assembly-based methods such as Cortex (Iqbal et al., 2012) and discoSnp++ (Peterlongo et al., 2017) form another line of research: the main idea of such tools consist in assembling the reads in a de Bruijn graph and then analyzing the bubbles in this graph to detect the variants. However, since read assembly is a computationally expensive task, such tools are still highly time consuming. Recent tools for genotyping and variant calling like Graphtyper (Eggertsson et al., 2017) and vg (Sirén, 2017), which are based on a graph representation of a pan-genome to avoid biases introduced by considering only the information included in a set of genomes (Computational Pan-Genomics Consortium, 2016), are nevertheless heavy in both computational space and time. Moreover, the size of indexes of variation graphs may be subjected to an exponential growth in the number of variants included, and indexes typically require a great deal of computational resources to be updated with newly discovered variants. When the task is to call the genotype in positions where variants have been previously annotated, alignment-free methods come to the aid. This is a typical case in a medical setting, where the discovery of new variants is not desired, but, rather, what is important is to know the genotype at certain loci that are already established to be of medical relevance.

Recent mapping-free genotyping tools such as LAVA (Shajii et al., 2016) and VarGeno (Sun and Medvedev, 2018) are word-based methods that, given a list of known SNP loci, call SNPs as either mutant or wild-type up to an order of magnitude faster than the usual alignment-based methods. A major shortcoming of these tools is the large memory requirement, which can easily exceed hundreds of GB of RAM. Their strategy is to create a dictionary for both the reference genome and the SNP list that maps each *k*-mer to the positions at which it appears and then to call variants from the reads by evaluating *k*-mers frequency. FastGT (Pajuste et al., 2017) is yet another *k*-mer-based method to genotype sequencing data: it strongly relies on a pre-compiled database of biallelic single nucleotide variants (SNVs) and corresponding *k*-mers, obtained by subjecting the *k*-mers that overlap known SNVs to several filtering steps. Such filters remove from the database the SNPs for which unique *k*-mers (i.e., not occurring elsewhere in the reference genome) are not observed, those that are closely located (i.e., that are less than *k* bases apart), and others; after the filtering steps, only 64% of biallelic SNVs survive and are therefore identifiable. These tools implement strategies to represent and analyze SNPs that improve the time performance but, on the other hand, do not allow to model indels and close variants.

Short insertions and deletions of nucleotides (*indels*) are believed to represent around 16%–25% of human genetic polymorphism (Mills et al., 2006). The presence of indels can be associated with a number of human diseases (Hasan et al., 2015, Ku et al., 2010, Mullaney et al., 2010): for instance, cystic fibrosis (Mullaney et al., 2010), lung cancer (Sequist et al., 2008), Mendelian disorders (MacArthur and Tyler-Smith, 2010), and Bloom syndrome (Kaneo et al., 1996) are all known to be closely correlated to indels. Indels are particularly challenging to call from NGS data, because mapping is more difficult when the reads overlap with indels (Montgomery et al., 2013).

Recently, mapping-free strategy was also applied to the discovery of *de novo* variants (Standage et al., 2019), i.e., variants that exist in a child but do not exist in both its parents.

In this paper we introduce MALVA, a rapid, lightweight, alignment-free method to genotype known (i.e., previously characterized) variants, including indels and close SNPs, in a sample of reads. MALVA is a word-based method: each allele of each known variant is assigned a *signature* in the form of a set of *k*-mers, which allows to efficiently model indels and close variants. The genotypes will be called according to the frequency of such signatures in the input reads. Based on the well-known Bayes' formula, we also design a new rule to genotype multi-allelic variants (i.e., variants such that more than one alternate allele is known): even if such variants are trickier to genotype than biallelic ones, we are still able to achieve high precision and recall, as revealed in the real-data experiments we conducted. MALVA directly analyzes a sample leveraging on the information of the variants included in a VCF file, which is the standard format released by the 1000 Genomes Project (Sudmant et al., 2015) (1KGP from now on). To the best of our knowledge, MALVA is the first mapping-free tool able to call indels. Moreover, it proved to be the only such tool capable of handling the huge number of variants included in the latest version of the VCF released by the 1KGP.

## Results

In this section we will describe the implementation details of MALVA and we will provide an experimental analysis on real data. All the analyses were performed on a 64-bit Linux (Kernel 4.4.0) system equipped with four 8-core Intel Xeon 2.30 GHz processors and 256 GB of RAM.

We performed an experimental analysis on real data to evaluate the real feasibility of our method, comparing MALVA to one mapping-free method, one assembly-based approach, and two different alignment-based pipelines. Among the mapping-free methods proposed in the literature we chose VarGeno, as it is an improved version of LAVA that provides better efficiency and accuracy (Sun and Medvedev, 2018). For completeness, we included in our evaluation discoSnp++(assembly-based) and the two most widely used alignment-based pipelines, denoted by BCFtools and GATK, respectively. The pipeline denoted by BCFtools consists of an alignment step performed with BWA-MEM (Li and Durbin, 2009) followed by a variant discovering step performed using BCFtools (Li, 2011). The latter consists of an alignment step performed with BWA-MEM and a variant discovering step performed with GATK (McKenna et al., 2010), as recommended by the *GATK Best Practices* (DePristo et al., 2011).

MALVA was run setting *k*_*s*_ equal to 47, *k*_*c*_ equal to 53, *ɛ* equal to 0.1%, and Bloom filters size equal to 8 GB and considering the genotype data and the *a priori* frequencies of the alleles of the EUR population, since the individual under analysis is part of it.

We tested the tools using the Illumina WGS dataset of the well-studied NA12878 individual provided by the Genome In A Bottle (GIAB) consortium (Zook et al., 2014). We chose this individual because the variant calls provided are highly reliable and can be effectively used to assess the precision and the recall of the considered methods. We downloaded the alignments of its 30x downsampled version and used SAMtools (Li et al., 2009) to extract the corresponding FASTQ file, obtaining 696,168,435 150-bp-long reads. As reference genome and set of known variants, we used the GRCh37 primary assembly and the VCF files provided by Phase 3 of the 1KGP (Sudmant et al., 2015). These VCF files contain a total of 84,739,838 variants, the phased genotype information of 2,504 individuals, and the *a priori* frequency of each allele of each variant of five populations. As stated earlier, from this VCF, in our evaluation MALVA extracted and considered only the individuals from the EUR population, for a total of 502 individuals. We note that we also removed the NA12878 individual from the input to better analyze MALVA and its capability in genotyping an unknown individual.

We note that VarGeno requires a different formatting of the fields describing the *a priori* frequencies of the alleles than the ones in the VCF file provided by the 1KGP. Thus, we formatted the input files as required before running VarGeno.

VarGeno could not complete the analysis of this dataset, from now on denoted by FullGenome, on our server. To test whether VarGeno crashed owing to excessive memory usage, we tried to run it on the same instance on a cluster with 1 TB of RAM, but nevertheless it could not complete the analysis, crashing after 20 min. To include VarGeno in our evaluation, we chose 12 chromosomes to create a smaller dataset, denoted by HalfGenome, that thus contains some half of the variants and the reads of the FullGenome dataset.

Each tool was evaluated in terms of variant calling accuracy and efficiency (wall time and memory usage). We note that some steps of the previously cited tools can use multiple threads to improve the time performance (namely, KMC3 for MALVA, discoSnp++, BWA-MEM for BCFtools and GATK, and the variant discovery steps of GATK). Whenever we had this choice, we provided four threads to each tool. We used hap.py (Krusche et al., 2019), the tool developed for the evaluation of variant callers in the recent *PrecisionFDA Truth Challenge* (https://precision.fda.gov/challenges/truth), and the/usr/bin/time system tool to gather the required data.

Table 1 shows the results obtained by the considered tools on both the FullGenome and the HalfGenome datasets. We point out that hap.py computes precision and recall considering only non-reference VCF records (i.e., non 0/0 calls). A qualitative representation of these results is available in Figure S1 of the Supplemental Information.

**Table 1:** Accuracy and Efficiency Results on the HalfGenome and FullGenome Datasets

Dataset	Tool	P_SNP_	R_SNP_	P_INDEL_	R_INDEL_	Time (hh:mm)	RAM (GB)
HalfGenome	MALVA	93.8%	91.1%	86.0%	81.4%	04:33	30
HalfGenome	VarGeno	97.5%	88.1%	39.5%	0.1%	02:31	52
HalfGenome	discoSnp++	89.5%	39.3%	80.8%	24.2%	07:45	7
HalfGenome	BCFtools	91.2%	94.8%	44.9%	55.4%	24:35	6
HalfGenome	GATK	91.7%	95.1%	53.2%	79.9%	34:43	32
FullGenome	MALVA	92.5%	90.0%	85.0%	80.6%	09:18	39
FullGenome	VarGeno	–	–	–	–	–	–
FullGenome	discoSnp++	86.9%	37.7%	80.0%	22.6%	14:19	9
FullGenome	BCFtools	91.6%	94.4%	44.7%	54.6%	54:23	9
FullGenome	GATK	92.1%	94.7%	53.2%	79.2%	73:36	33

For each dataset, we reported the values of Precision (P) and Recall (R) obtained by the considered tools on both SNPs and indels. The efficiency results are shown in terms of wall clock time and peak memory usage. VarGeno could not complete the analysis of the FullGenome dataset; thus, we did not report its results on this dataset. See also Figure S1.

**Figure 1:**
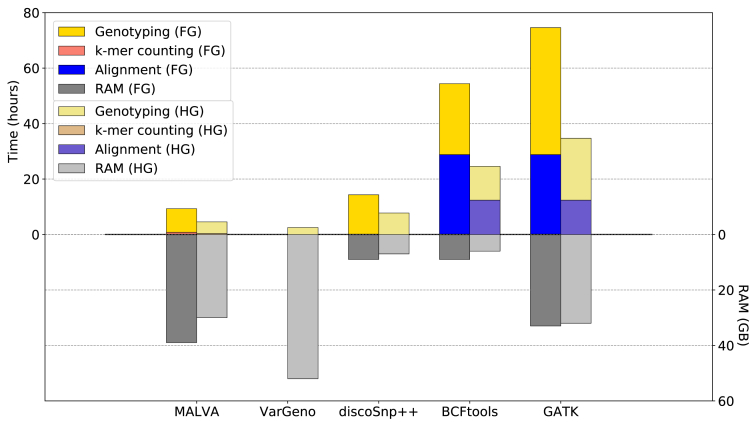
Time and RAM Required by Each Tool to Analyze Both Datasets The running times are partitioned by steps performed, whereas the RAM usage represents the peak memory of the entire process. For ease of presentation, we denoted the FullGenome dataset as FG and the HalfGenome dataset as HG. Note that we did not include VarGeno running time and RAM usage on the FullGenome dataset since it crashed after 20 min.

As expected, MALVA, VarGeno, and discoSnp++ are faster than the tested alignment-based approaches, i.e., BCFtools and GATK. Indeed, MALVA, VarGeno, and discoSnp++ required 4.5, 2.5, and 7.5 h to analyze the HalfGenome dataset, respectively, whereas BCFtools and GATK required 24.5 and 34.5 h. We note that half of the time required by BCFtools and one-third of the time required by GATK was spent running BWA-MEM, which completed its task in 12.5 h (using four threads). The same trend applies to the analysis of the FullGenome dataset, on which each tool required roughly twice the time required on the HalfGenome dataset. A qualitative representation of the running time and the memory usage of each tool is shown in Figure 1.

For what concerns the memory usage, BCFtools proved to be the least memory intensive approach, requiring less than 10 GB of RAM on both datasets to map the reads and less than 1 GB of RAM to call the variants. MALVA and GATK showed similar memory requirements, with GATK showing almost no difference between the two analyses and MALVA increasing the memory consumption by only 23% for the bigger dataset. VarGeno required slightly less than twice the amount of memory required by MALVA on the HalfGenome dataset.

Precision and recall of all the tools varied little over the two datasets, proving that the number of variants and reads only slightly affects their accuracy. As expected, BCFtools and GATK achieved the best recall for non-homozygous reference SNPs owing to the mapping step, which provides a more precise coverage of the alleles and allows to better discern repeated regions of the reference genome. discoSnp++ achieved the lowest recall, whereas VarGeno obtained 3% less recall than MALVA, which in turn called correctly 91.1% of the SNPs. On the other hand, MALVA, discoSnp++, BCFtools, and GATK achieved comparable precision on SNPs, whereas VarGeno obtained the highest one. Overall, on non-homozygous reference SNPs, VarGeno seems to be the most conservative tool among those tested, as it prefers not to call SNPs when there is any uncertainty. On the contrary, MALVA, in avoiding the loss of any potentially interesting information, deliberately prefers to detect any potential alternate allele in the donor, at the cost of a slight loss in precision.

**Figure 2:**
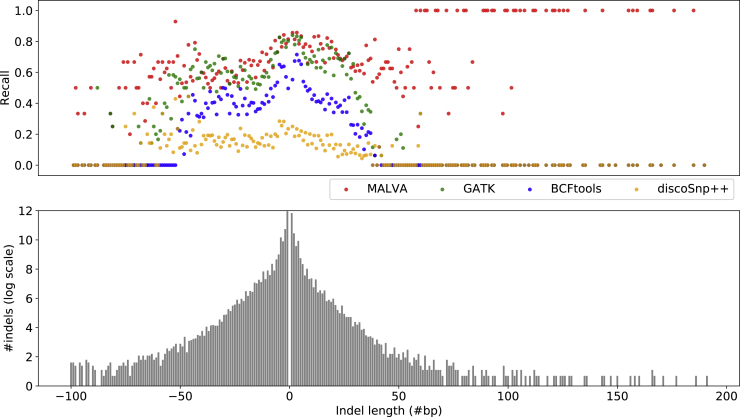
Influence of Indel Size on the Recall Achieved by the Four Considered Tools on the FullGenome Dataset The histogram shows the frequency distribution (on logarithmic scale) of the indels with respect to their length. The scatterplot shows the recall of the tools with respect to the indel size.

Remarkably, on indels MALVA obtained significantly better recall than BCFtools and discoSnp++ and better precision than any other tool. As expected, since the method of VarGeno is not designed to manage indels, it was only able to genotype a negligible percentage of them. On the other hand, discoSnp++ achieved a high precision but it was only able to call less than a quarter of the total indels. Finally, BCFtools showed a very low precision and recall on indels, whereas GATK achieved a recall similar to MALVA but a low precision. The low precision achieved by the alignment-based tools is mainly due to the difficulties in aligning reads that overlap with indels. We also performed a more detailed analysis on the influence of indel size on the recall obtained by the tools on the FullGenome dataset. As shown in Figure 2, MALVA proved to be the only tool able to call long indels (more than ∼40/50 bases), whereas the other tools are limited to short indels (that are also the most common ones). In any case, MALVA outperformed the other tools even on these shorter indels. We did not include VarGeno in this analysis since its recall on indels was lower than 1% even on the HalfGenome dataset.

Overall, MALVA proved to be an accurate and efficient alternative to mapping-based pipelines for variant calling, achieving good results both on SNPs and indels. The experimental evaluation shows the usefulness of the formalization of signature of an allele, of the extension to multi-allelic SNPs and indels, and of the ability to manage variants in dense genomic regions. A more in-depth comparison of MALVA and VarGeno is provided in the next section.

### Comparison of MALVA and VarGeno Output

**Figure 3:**
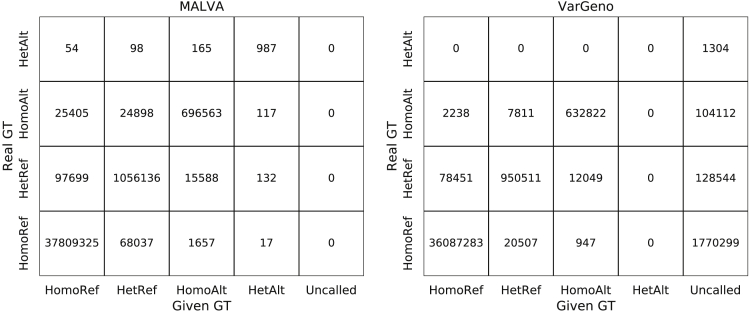
Comparison between Real Genotype (Provided by the 1000 Genomes Project) and Genotype Called by MALVA and VarGeno HomoRef stands for Homozygous Reference, HetRef stands for Heterozygous Reference, HomoAlt stands for Homozygous Alternate, HetAlt stands for Heterozygous Alternate, and Uncalled means that the given variant was not called by the tool.

To assess whether the mapping-free approaches under analysis produce some systematic error, we considered the HalfGenome dataset and performed a more in-depth analysis of the SNPs genotyped by the two tools. For each tool, Figure 3 reports the number of correct genotypes output, grouping them in *homozygous reference* (i.e., 0|0), *heterozygous reference* (i.e., 0|1, 0|2, and so on), *homozygous alternate* (i.e., 1|1, 2|2, and so on), and *heterozygous alternate* (i.e., 1|2, 1|3, 2|3, and so on). As stated in the previous section, we recall that the precision and recall output by hap.py do not consider homozygous reference genotypes; thus, the analysis we present in this section allows us to better understand the behavior of the tools. Since VarGeno is not able to manage indels, we decided to not include them in this analysis.

Consistently with the precision and recall results of hap.py, MALVA detects between 5% and 10% more correct variants than VarGeno in all classes, at the cost of producing more erroneous calls. We note that overall VarGeno filters out 2,004,259 of the 39,796,878 SNPs in the truth.

**Figure 4:**
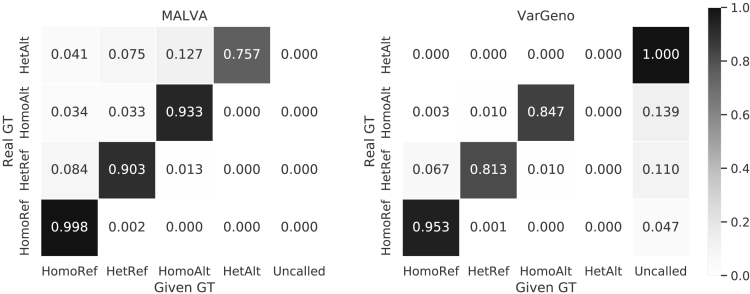
Comparison between Real Genotype (Provided by the 1000 Genomes Project) and Genotype Called by MALVA and VarGeno, Normalized by Rows HomoRef stands for Homozygous Reference, HetRef stands for Heterozygous Reference, HomoAlt stands for Homozygous Alternate, HetAlt stands for Heterozygous Alternate, and Uncalled means that the given variant was not called by the tool.

Both tools show a similar pattern in erroneous calls. More precisely, erroneously genotyped homozygous reference variants were mostly genotyped as heterozygous reference and, vice versa, erroneously genotyped heterozygous reference variants were mostly genotyped as homozygous reference. On the other hand, erroneous homozygous alternate variants in the donor were mostly genotyped as heterozygous reference by VarGeno, whereas MALVA evenly distributed the errors between homozygous reference and heterozygous reference calls. Finally, erroneous heterozygous alternate variants in the donor were mostly genotyped as homozygous alternate variants by MALVA, meaning that the method proposed in this paper was able to detect the fact that the allele was not the reference allele but it called one of the two alternate alleles of the donor erroneously. Figure 3 shows a comparison between real genotype (provided by the 1000 Genomes Project) and genotype called by MALVA and VarGeno on SNPs. Figure 4 shows the same data row-normalized.

Overall, the errors produced by both tools were “partial” errors in the sense that they rarely mis-call both alleles of the donor.

## Discussion

In this article, we presented MALVA, the first efficient mapping-free genotyping tool that is able to handle multi-allelic variants and indels. We compared MALVA with VarGeno, the state-of-the-art mapping-free genotyping tool, and showed that our method is less memory intensive, achieves better recall, handles dozens of millions of variants effectively, and provides correct genotypes even for indels. We also compared our tool with two variant discovery pipelines, namely, GATK and BCFtools, showing that MALVA is an order of magnitude faster while achieving better accuracy on indels and similar accuracy on SNPs.

MALVA proved to be able to efficiently manage a huge amount of variants like those provided by the 1000 Genome Project (about 80 million variants) and to handle multi-allelic variants and indels. These fundamental features allow our method to exploit the whole information in input, without filtering out any data that might be crucial in successive analyses. Most notably, MALVA's ability to genotype indels allows one to apply mapping-free techniques to many clinical contexts, including screens for genetic predispositions for disease linked to the presence of indels (Rimmer et al., 2014, Warren et al., 1987).

Future steps will be devoted to improving the efficiency of MALVA by exploiting the parallel architecture of modern machines and to extending the method to genotype trios. Another possible future direction consists in designing a mapping-free method for genotyping known variants using long reads such those produced by the latest PacBio and Oxford Nanopore technologies. Indeed, MALVA is specifically designed for dealing with Illumina short-read data and cannot be directly applied to long-read data owing to their higher error rate.

### Limitations of the Study

MALVA achieves high accuracy and efficiency by analyzing only a subset of the sequences in input, i.e., the *k*-mers centered on the alleles. Although using the concept of signature proved to be effective in this context, in some edge cases two alleles might share the same signature and our method will not be able to discern between the two. A simple solution to reduce the occurrences of different alleles sharing the same signature is to increase the value of *k*. Unfortunately, increasing *k* beyond 40–50 has two main drawbacks: (1) it is computationally expensive and (2) owing to errors, the probability that such *k*-mers appear in the input reads decreases. To face this limitation, future works should (1) investigate the effect of using multiple *k*-mers spanning each allele and (2) exploit the *k*-mers flanking the potential occurrence of an allele in the read.

## Methods

All methods can be found in the accompanying Transparent Methods supplemental file.

## Supplemental Information

Document S1. Transparent Methods and Figures S1–S3
