# Supplementary material for: MALVA: Genotyping by Mapping-free ALlele Detection of Known VAriants
Source: iScience. 2019 Jul 12;18:20–7. doi: 10.1016/j.isci.2019.07.011 (PMC6664100; doi:10.1016/j.isci.2019.07.011)
Supplement: Document S1. Transparent Methods and Figures S1–S3 [file mmc1.pdf]

ISCI, Volume *18*

Supplemental Information

MALVA: Genotyping by Mapping-free ALlele Detection of Known VAriants

Luca Denti, Marco Previtali, Giulia Bernardini, Alexander Schönhuth, and Paola Bonizzoni

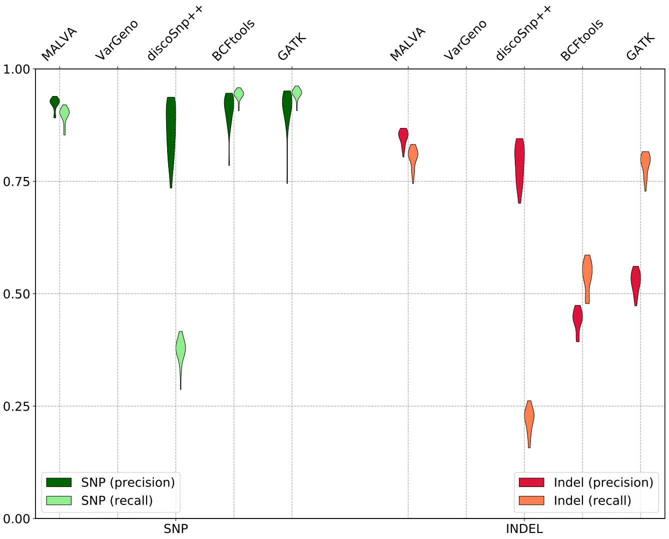

(a) `FullGenome` dataset

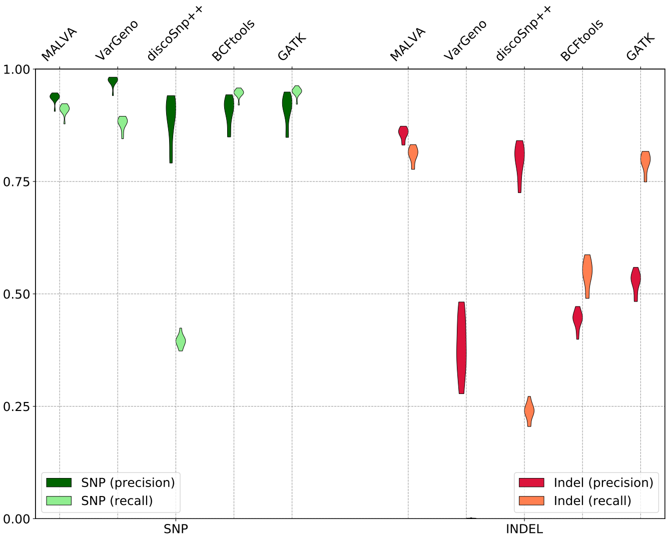

(b) `HalfGenome` dataset

Figure S1: Qualitative representation of the accuracy results, Related to Table 1. Each violin plot represents the precision and the recall (computed with `hap.py`) achieved by the considered tools on both SNPs and indels. This is a qualitative representation of the information summarized by the table in the main document.

S1. Transparent Methods

We will first introduce some preliminary definitions and then we will describe the approach we propose (MALVA) for the mapping-free genotyping of known variants.

S1.1. Preliminaries

Let Σ be an ordered and finite alphabet of size σ and let $t = c_1, \ldots, c_k$, where $c_j \in \Sigma$ for $j = 1, \ldots, k$, be an ordered sequence of k characters drawn from Σ, we say that t is a k-mer. When a k-mer originates from a double stranded DNA, it is common to consider it and its reverse-complemented sequence as the same k-mer, and to say that the one that is lexicographically smaller among the two is the *canonical* one. In the following, we will abide by this definition and whenever we refer to a k-mer we implicitly refer to its canonical form. Moreover, to avoid k-mers being equal to their reverse-complement, we will only consider odd values of k.

A Bloom filter (Bloom, 1970) is a probabilistic space-efficient data structure that represents a set of elements and allows approximate membership queries. The result of such queries may be a false positive but never a false negative. Bloom filters are usually represented as the union of a bitvector of length m and a set of h hash functions $\{\mathtt{H}_1, \ldots, \mathtt{H}_h\}$, each one mapping one element of the universe to one integer in $\{1, \ldots, m\}$. Using these data structures, the addition of an element e to the set is performed by setting to 1 the bitvector's cells in positions $\{\mathtt{H}_1(e), \ldots, \mathtt{H}_h(e)\}$, while testing if an element is in the set boils down to checking whether the same positions are all set to 1. Due to collisions of the hash functions, an element can be reported as present in the set even if it is absent. Nevertheless, the false positive rate of a Bloom filter of a set of n elements, with h hash functions and an array of m bits is $(1 - e^{\frac{hn}{m}})^h$; therefore, to increase the size of the Bloom filter decreases the false positive rate. Due to their simplicity and efficiency, Bloom filters have been applied to multiple problems in bioinformatics, such as representing de Bruijn graphs (Chikhi and Rizk, 2013) and counting k-mers in a sample (Melsted and Pritchard, 2011).

Let B be a bitvector, the $\texttt{rank}_1$ function reports, for each position $i \in \{1, \ldots, |B| + 1\}$, the number of 1s from the beginning of B to i (excluded); we refer to such value as $\texttt{rank}_1(i, B)$. Clearly, $\texttt{rank}_1(i, B)$ is not defined for $i \leq 0$ and for $i > |B|+1$, $\texttt{rank}_1(1, B)$ is 0, and $\texttt{rank}_1(|B|+1, B)$ is the number of 1s in B. By using succinct support data structures and by a linear time preprocessing step, it is possible to answer $\texttt{rank}_1$ queries in constant time for any position of the bitvector (Vigna, 2008).

The difference between the genetic sequence of two unrelated individual of the same species is estimated to be smaller than 0.1% (Venter et al., 2001); therefore, it is common to represent the DNA sequence of an individual as a set of differences from a *reference* genome. Indeed, thorough studies (Consortium et al., 2015, 2003; consortium et al., 2015) of the variations across different individuals encode such information as a `VCF` (Variant Calling Format) file (Danecek et al., 2011). In the following, we will call *variant* the information encoded by a data line of a `VCF` file. Besides the genotype data, we are interested in the information carried by the second, fourth, fifth, and eighth field of a `VCF` line, namely: (i) field `POS` that is the position of the variant on the reference, (ii) field `REF` that is the reference allele starting in position `POS`, (iii) field `ALT` that is a list of alternate alleles that in some sample replace the reference allele, and (iv) field `INFO` that is a list of additional information describing the variant. From the latter list we will get the frequencies of reference and alternate alleles, which are needed to call the genotype of a given individual. We denote with $\texttt{POS}(v)$, $\texttt{REF}(v)$, $\texttt{ALT}(v)$, $\texttt{FREQ}(v)$, and $\texttt{GTD}(v)$ the reference position, reference allele, list of alternate alleles, list of allele frequencies, and genotype data of a variant v, respectively. The variants we take into account are SNPs (*i.e.*, both `REF` and all the elements of `ALT` are single base nucleotides) and indels (`REF` and at least one element of `ALT` are not of the same length). Moreover, given an allele a (either reference or alternate) of some variant v, we refer to its sequence of nucleotides as $\texttt{SEQ}(a)$, *i.e.*, $\texttt{SEQ}(a)$ is the string that represents a.

Let R be a reference genome and let `V` be a `VCF` file that describes all the known variants of R. Since the genotype data provides information on the

alleles expressed in each genome, another way of thinking of a VCF file is as an encoding of a set of genomes $\mathcal{G}$. Each haplotype of the genomes in $\mathcal{G}$ can be reconstructed by modifying R according to the genotype information associated to each variant. For ease of presentation, in the following we use the term genome and haplotype interchangeably, although each genome of a polyploid organism is composed of multiple haplotypes.

Let $\mathcal{G}$ be the set of genomes encoded by a VCF file and let a be an allele of some variant v, we denote by $\mathcal{G}^a \subseteq \mathcal{G}$ the subset of genomes that include a. We say that a variant v is *k-isolated* if there is no other known variant within a radius of $\lfloor k/2 \rfloor$ from the center of any of its alleles, as formally stated in the following definition.

Definition 1 (k-isolated variant). A variant v is k-isolated if, for all $a \in \texttt{ALLELES}(v)$ and $g \in \mathcal{G}^a$, there is no variant $v' \neq v$ with an allele $a' \in \texttt{ALLELES}(v')$ such that $g \in \mathcal{G}^{a'}$ and either $|\texttt{BEGIN}_g(a') - \texttt{CENTER}_g(a)| \leq \lfloor k/2 \rfloor$ or $|\texttt{CENTER}_g(a) - \texttt{END}_g(a')| \leq \lfloor k/2 \rfloor$, where $\texttt{ALLELES}(v) = \texttt{REF}(v) \cup \texttt{ALT}(v)$, $\texttt{BEGIN}_g(a)$ is the position of the first base of a in g, $\texttt{END}_g(a)$ the position of the last base, and $\texttt{CENTER}_g(a)$ the position of the $\lceil \frac{|a|}{2} \rceil$-th base of a in g.

The procedure we will present in the next section is heavily based on the concept of *signature* of an allele. Intuitively, a signature of the allele a of a variant v is the k-mer centered in a in some genome g in $\mathcal{G}^a$. Note that, depending on the genomes encoded by the VCF file (specifically, if variants less than k bases apart are known), an allele might have multiple signatures. Moreover, if $\texttt{SEQ}(a)$ is longer than k bases, the previous definition is not well formed, since there is no k-mer that can be centered in a. In this case, we define the signature of a as the set of its substrings of length k. The following definition formalizes the notion of signature of an allele.

Definition 2 (Signature of an allele). Let $\mathcal{G}$ be the set of all the genomes encoded by a VCF file V and let k be an odd positive value. Let v be a variant in V, let a be one of the alleles of v, and let $\mathcal{G}^a \subseteq \mathcal{G}$ be the set of the genomes that include a. If $\texttt{SEQ}(a)$ is longer than k bases, we say that the signature of a

	1	2	3	4	5	6	7	8	9	10	11	12	13
$\mathcal{R}$	A	G	A	T	C	C	T	G	C	G	A	A	G

	**Pos**	**Ref**	**Alts**	**Donors**		
v_1	5	C	AAA	0|1	0|0	1|1
v_2	7	T	G	0|1	0|1	0|1
v_3	10	G	A,C	0|0	1|2	2|0

Variant	Allele	Signatures
v_2	$a_0 = \text{T}$	{ {TCC**T**GCG}, {TCC**T**GCA}, {AAC**T**GCC} }
	$a_1 = \text{G}$	{ {AAC**G**GCG}, {TCC**G**GCC} }

Figure S2: Signatures of the alleles of variant v_2. $\mathcal{R}$ is the reference sequence and the table on the right is a VCF information associated to it, representing 3 variants: an indel (v_1), a bi-allelic SNP (v_2), and a multi-allelic SNP (v_3). The last columns of the VCF file carry the genotype information of 3 individuals. The table at the bottom reports the signatures of each allele of variant v_2. Note that there are only 5 signatures although 6 haplotypes are encoded by the VCF file since the second haplotype of the first and third individual are the same. We highlighted in red the genotype information associated to the second haplotype of the second genome and the corresponding signature.

is the set of all the substrings of length k of $\mathtt{SEQ}(a)$. If $\mathtt{SEQ}(a)$ is shorter than k bases, we say that $\{x\mathtt{SEQ}(a)y\}$ is the signature of a in a genome g in $\mathcal{G}^a$ if: (i) $x\mathtt{SEQ}(a)y$ is a k-mer, (ii) $|x| = \lfloor \frac{k-|\mathtt{SEQ}(a)|}{2} \rfloor$, (iii) $|y| = \lceil \frac{k-|\mathtt{SEQ}(a)|}{2} \rceil$, (iv) x is a suffix of the sequence that precedes a in g, and (v) y is a prefix of the sequence that follows a in g.

We will refer to the set of all the possible signatures of an allele a as $\mathtt{SIGN}(a)$ and we say that k is the *length of the signature*. An example of signatures of an allele is shown in Figure S2. Notice that the same k-mer may appear in the signature of more than one allele.

In the following we will leverage on the definition of signature of an allele to detect its presence in an individual without mapping the reads to the reference genome. More precisely, we will analyze whether the k-mers of a given signature are present in the reads and use such information as an *hint* of the presence of the allele. Unlike other approaches (Pajuste et al., 2017), Definition 2 admits the presence of the alleles of multiple variants in a single signature, allowing MALVA to manage variants that are not k-isolated. Indeed, the set of signatures of an allele represents all the genomic regions where the allele appears in the genomes encoded by the VCF file.

S1.2. MALVA's approach

In this section we will describe MALVA, the method we designed to genotype a set of known variants directly from a read sample. The general idea of MALVA is to use the frequencies of the signatures of a variant in the sample to call its genotype. The method works under the assumption that given a sample of reads from a genome with standard coverage depth, if an allele is included in the genome then at least one of its signatures must exist as substrings in multiple reads (depending on the coverage depth and the length of the signature). We leverage on this concept to genotype known variants directly from the input reads.

MALVA takes as input a reference genome, a VCF file representing all its known variants, and a read sample; it outputs a VCF file containing the most probable genotype for each variant. The main method is composed of four steps.

In the first step, MALVA computes the set of signatures of length k_s of all the alternate alleles of all the variants in VCF and stores them in the set ALTSIG. In the same step, the signatures of the reference alleles are computed and stored in a second set named REFSIG. For each k_s-mer t of a signature s two weights, one representing the number of occurrences of t in an alternate allele signature and one representing the number of occurrences of t in a reference allele signature, are stored. We will refer to these two values as $w_t^{\mathtt{A}}$ and $w_t^{\mathtt{R}}$, respectively.

We note that for small values of k_s the probability that the k_s-mers that constitute a signature appear in other regions of the genome is high. Since in the following steps MALVA exploits the signatures' sets of the alleles of each variant to call the genotypes, the presence of conserved regions of the reference genome identical to some signature could lead the tool to erroneously genotype some variants. To get rid of a large amount of wrong calls, in the second step MALVA makes use of the context around the allele to distinguish its signatures from such regions. More precisely, if a k_s-mer of a signature of an alternate allele appears somewhere in the reference genome, MALVA extracts the context of length k_c (with $k_c > k_s$) covering the reference genome region and collects such k_c-mers in a third set (REPCTX).

In the third step, MALVA extracts all the k_c-mers from the sample along with the number of its occurrences. For each k_c-mer t_c that occurs w times in the sample, the k_s-mer t_s that constitutes the center of t_c is extracted. If t_s is found in REFSIG, $w^R_{t_s}$ is increased by w. Moreover, if t_c is not found in REPCTX and if t_s is in ALTSIG, $w^A_{t_s}$ is increased by w. Otherwise, if t_c is in REPCTX, $w^A_{t_s}$ is not updated since, although its central k_s-mer is identical to some k_s-mer of a signature of an alternate allele of some variant, it is indistinguishable from another region of the genome not covering the variant. We note that when $w^A_{t_s}$ is not updated, our method might miss a variant in the donor and report a false negative, although for large values of k_c this would rarely occur. The rationale behind this choice is to avoid biases due to k_c-mers in conserved regions of the reference genome, preferring not to include an alternate allele in the output whenever ambiguities arise.

Finally, in the fourth step, MALVA uses the weights computed in the previous step to call the genotypes.

In the rest of this Section we will detail each one of the four steps of MALVA.

Signature computation. The first step consists of building the signatures of the alleles of all the variants and adding them either to ALTSIG, if they are the signatures of an alternate allele, or to REFSIG, if they are the signature of the reference allele. If a variant v is k_s-isolated, we build $1+|\texttt{ALT}(v)|$ signatures, one for each allele of v. Otherwise, there are some genomes in $\mathcal{G}$ in which there is at least another allele of a variant that lays within a radius of $\lfloor k_s/2 \rfloor$ nucleotides from the center of the allele of v. In practice, this means that we have to look at the genotype data of the variants within such radius: for each allele a of v we reconstruct the k_s bases long portions of the genomes in $\mathcal{G}^a$ that constitute the signatures of a.

As pointed out in Definition 2, if $|\texttt{SEQ}(a)| \geq k_s$, the signature of a is the set of k_s-mers that appear in $\texttt{SEQ}(a)$. In this case we extract all such k_s-mers and add them either to REFSIG or ALTSIG. Otherwise, if $|a| < k_s$, we build the k_s bases long substrings of each genome in $\mathcal{G}^a$ centered in a by scanning the VCF

file and reconstructing the sequences according to the genotype information it includes. More precisely, let a be an allele of a variant v and let $V = \{v_1, \ldots, v_n\}$ be the set of variants such that, for all $1 \leq i \leq n$: (i) $v_i \neq v$, (ii) there exists an allele a_j in $\texttt{ALLELES}(v_i)$ such that a and a_j are both included in some genome g, and (iii) either ($\texttt{END}(a_j) < \texttt{BEGIN}(a)$ and $\texttt{CENTER}(a) - \lfloor k_s/2 \rfloor \leq \texttt{END}(a_j)$) or ($\texttt{END}(a) < \texttt{BEGIN}(a_j)$ and $\texttt{CENTER}(a) + \lfloor k_s/2 \rfloor \geq \texttt{BEGIN}(a_j)$) in g.

Given a, we use the genotype information stored in the VCF file to retrieve the haplotypes in which it is included, *i.e.*, a subset of the haplotypes in $\mathcal{G}^a$, and build the set V. Using V we gather all the alleles that precede and succeed a in the selected haplotypes and we use them, together with the reference sequence, to reconstruct *on the fly* the k_s-mer that covers a, by interposing reference substrings and allele sequences. Doing so, we don't need to reconstruct the whole haplotypes but we only analyze and reconstruct the required k_s-mers when needed.

Once all the k_s-mers have been constructed, they are added to REFSIG if a is the reference allele, to ALTSIG if it is an alternate allele.

Detection of repeated signatures. This step is aimed to detect and store in set REPCTX all the k_c-mers of the reference sequence whose central k_s-mer is included in some signature of some alternate allele, $k_c > k_s$. REPCTX will be used in a further step to discard alternate alleles that might be erroneously reported as expressed by MALVA only because they cannot be told apart from other identical regions of the reference sequence. To compute REPCTX, we extract all the k_c-mers of the reference sequence and test whether their central k_s-mer is in ALTSIG. If so, we add the k_c-mer to REPCTX to report that the k_s-mer is indistinguishable from some k_s-mer that is included in the signature of an alternate allele. The set REPCTX is then used in the next step as illustrated below. An example comprising the first two steps is shown in Section S1.3.

Alleles' signatures weights computation. In the third step, MALVA computes how many times the k_s-mers of each signature appear in the dataset. First, MALVA extracts all the k_c-mers of the read sample and tests their existence in REPCTX to

check whether their central k_s-mer cannot be told apart from some repetition in the reference genome. Then, given a k_c-mer t_c that occurs w times in the read sample, the k_s-mer t_s that constitutes its center is extracted. If t_s is found in `REFSIG`, *i.e.*, t_s is the signature of the reference allele of some variant, the weight $w^R_{t_s}$ is increased by w. Moreover, if t_c is not found in `REPCTX` and t_s is in `ALTSIG`, *i.e.*, k_s-mer t_s is uniquely associated to an alternate allele of some variant, the weight $w^A_{t_s}$ is increased by w. Conversely, if t_c is in `REPCTX`, $w^A_{t_s}$ is not updated. The last scenario happens when t_s is identical to the signature of an alternate allele of some variant (indeed, t_s is in `ALTSIG`), but even the enlarged context t_c (and consequently t_s) appears somewhere else in the reference genome.

Genotype calling. In the last step, `MALVA` uses the allele frequencies stored in the `INFO` field of the `VCF` file and the weights of the signatures computed in the previous step to call the genotype of each variant. To this aim, we extend the approaches proposed in the literature for bi-allelic variants (specifically, the one introduced in `LAVA` (Shajii et al., 2016)) to multi-allelic variants. While the approaches designed for genotyping bi-allelic variants only need to compute the likelihood of three genotypes, our technique must consider a larger number of possible genotypes.

Let v be a variant with $n-1$ alternate alleles. The number of possible distinct genotypes is $\binom{n}{2} + n = \frac{n(n+1)}{2}$, that is one *homozygous reference* genotype, $\binom{n}{2}$ *heterozygous* genotypes, and $n-1$ *homozygous alternate* genotypes. We will refer to the homozygous reference genotype as $G_{0,0}$, to the heterozygous genotypes as $G_{i,j}$ with $0 \leq i < j \leq n-1$, and to the homozygous alternate genotypes as $G_{i,i}$ with $1 \leq i \leq n-1$. Following well-established techniques (Shajii et al., 2016; McKenna et al., 2010; Li, 2011), we compute the likelihood of each genotype $G_{i,j}$ by means of the Bayes' theorem. Given the observed coverage C, we compute the posterior probability of each genotype as:

$$P(G_{i,j}|C) = \frac{P(G_{i,j})P(C|G_{i,j})}{P(C)}$$

that, by the law of total probability, can be expressed as:

$$P(G_{i,j}|C) = \frac{P(G_{i,j})P(C|G_{i,j})}{\sum_{p=0}^{n-1}\sum_{q=p}^{n-1} P(G_{p,q})P(C|G_{p,q})}$$

To calculate this probability, we compute the *a priori* probabilities of each genotype $G_{i,j}$ ($P(G_{i,j})$) and the *conditional probability* of the observed coverage given the considered genotype ($P(C|G_{i,j})$). The Hardy-Weinberg equilibrium equation ensures that for each variant v, $(\sum_{i=0}^{n-1} f_i)^2 = 1$, where $f_i = \texttt{FREQ}(v)[i]$, *i.e.*, the frequency of the i-th allele of v. We recall that $\texttt{FREQ}(v)$ is stored in the `INFO` field of the `VCF` file. The *a priori* probability of each genotype $G_{i,j}$ is therefore computed as follows:

$$P(G_{i,j}) = \begin{cases} f_i^2 & \text{if } i = j \\ 2f_i f_j & \text{otherwise} \end{cases}$$

To compute the conditional probability $P(C|G_{i,j})$, it is first necessary to compute the *coverages* of the alleles of the variant. Without loss of generality, let a_0 be the first allele of the variant, *i.e.*, a_0 is the reference allele with index 0. We recall that $\texttt{SIGN}(a_0)$ is the set of signatures of allele a_0 and that each signature is a set of one or more k-mers. We also recall that, in the previous step, for each k-mer t that belongs to some signature we computed two weights, namely w_t^R and w_t^A. Given a signature $s \in \texttt{SIGN}(a_0)$, we define its weight as the mean of the weights associated to the k-mers it contains, *i.e.*, $\frac{\sum_{t\in s} w_t^R}{|s|}$ where $|s|$ denotes the number of k-mers contained in signature s. Since the same allele may exhibit more signatures, we define the coverage c_0 of allele a_0 as the maximum value among the weights of its signatures, *i.e.*, $\max\{\frac{\sum_{t\in s} w_t^R}{|s|} : s \in \texttt{SIGN}(a_0)\}$. This formula can be easily modified to compute the coverage of an alternate allele (c_i for $i \geq 1$) by switching w_t^R with w_t^A. The coverage c_i of an allele a_i of a variant is thus computed as follows:

$$c_i = \begin{cases} \max\{\frac{\sum_{t\in s} w_t^R}{|s|} : s \in \texttt{SIGN}(a_0)\} & \text{if } i = 0 \\ \max\{\frac{\sum_{t\in s} w_t^A}{|s|} : s \in \texttt{SIGN}(a_i)\} & \text{otherwise} \end{cases}$$

By extending the approach adopted in (Shajii et al., 2016), we consider each $P(C|G_{i,j})$ to be multinomially distributed. Given a homozygous genotype $G_{i,i}$,

we assume to observe the i-th allele, which is the correct one, with probability $1-\varepsilon$ (where ε is the expected error rate) whereas the other $n-1$ alleles (the erroneous ones) with probability $\frac{\varepsilon}{n-1}$ each. Hence, we compute the conditional probability of an homozygous genotype as:

$$P(C|G_{i,i}) = \binom{c_i + C_E}{c_i}(1-\varepsilon)^{c_i}\left(\frac{\varepsilon}{n-1}\right)^{C_E}$$

where C_E is the total sum of the coverages of the erroneous alleles, *i.e.*, $C_E = \sum_{j\in\{0,\ldots,n-1\}\setminus\{i\}} c_j$. For what concerns heterozygous genotypes, we assume to observe the correct alleles, *i.e.*, the i-th and the j-th allele, with equal probability $\frac{1-\varepsilon}{2}$ whereas the other $n-2$ erroneous alleles with probability $\frac{\varepsilon}{n-2}$ each. We compute the conditional probability of an heterozygous genotype as follows:

$$P(C|G_{i,j}) = \binom{c_i + c_j + C_E}{c_i + c_j}\binom{c_i + c_j}{c_i}\left(\frac{1-\varepsilon}{2}\right)^{c_i}\left(\frac{1-\varepsilon}{2}\right)^{c_j}\left(\frac{\varepsilon}{n-2}\right)^{C_E}$$

where, again, C_E is the sum of the coverages of the erroneous alleles, *i.e.*, $C_E = \sum_{p\in\{0,\ldots,n-1\}\setminus\{i,j\}} c_p$.

Finally, after computing the posterior probability of each genotype, `MALVA` outputs the genotype with the highest likelihood.

S1.3. Example of k-mers weight computation

In this section we present an example of computation of the weights associated with the signatures' k_s-mers. Figure S3 shows an example composed of three variants and two reads. In this example the values of k_s and k_c are set to 7 and 11, respectively. Subfigure (a) shows the 26-bases long reference sequence. Subfigure (b) reports on the left two bi-allelic variants (v_1 and v_2) and one multi-allelic variant (v_3), and on the right the signatures of each allele of v_2. Subfigure (c) shows the elements of `ALTSIG` and `REFSIG` related to v_2. We note that the second signature in `ALTSIG` is composed of a single k_s-mer (t_s, equal to `TCCGGCG`) that appears in the reference genome, starting from position 17. Thus, the k_c-mer starting in position 15 and ending in position 25 (t_c, equal to `GATCCGGCGAA`) is added to `REPCTX`. Subfigure (d) shows two 11-bases long reads including t_s, extracted from position 3 and 15 of the donor. Clearly, only

(*a*)

	1	2	3	4	5	6	7	8	9	10	11	12	13	14	15	16	17	18	19	20	21	22	23	24	25	26
$\mathcal{R}$	A	T	A	T	C	C	T	G	C	G	T	A	G	A	G	A	**T**	**C**	**C**	**G**	**G**	**C**	**G**	A	A	G

(*b*)

	**Pos**	**Ref**	**Alts**	**Donors**		
v_1	5	C	AAA	0|1	0|0	1|1
v_2	7	T	G	0|1	0|1	0|1
v_3	10	G	A,C	0|0	1|0	2|0

Variant	Allele	Signatures
v_2	a_0 = T	{ {TCC**T**GCG}, {TCC**T**GCA} , {AAC**T**GCC} }
	a_1 = G	{ {AAC**G**GCG}, {TCC**G**GCG}}

(*c*)

ALTSIG
{AAC**G**GCG}
{TCC**G**GCG}

REFSIG
{TCC**T**GCG}
{TCC**T**GCA}
{AAC**T**GCC}

REPCTX
GATCCGGCGAA

(*d*)

	1	2	3	4	5	6	7	8	9	10	11
r_1	T	A	T	C	C	G	G	C	G	T	A
r_2	G	A	T	C	C	G	G	C	G	A	A

Figure S3: Example with 3 variants and two reads. Subfigure (*a*) shows a reference genome of 26 bases, Subfigure (*b*) reports 3 variants and the signatures of each allele of variant v_2, Subfigure (*c*) reports the subsets of `ALTSIG`, `REFSIG`, and `REPCTX` including the elements related to v_2, and Subfigure (*d*) presents two reads of length 11.

r_1 should contribute to the detection of the alternate allele of v_2 in the donor, since r_2 was sequenced from another position of the genome (*i.e.*, $w^A_{t_s}$ should be equal to 1 in this case). To this aim, `REPCTX` comes to an aid; indeed, when analyzing r_1 the k_c-mer covering t_s is extracted (*i.e.*, the whole read) and its inclusion in `REPCTX` is tested. Since `TATCCGGCGTA` is not in `REPCTX` and t_s is in `ALTSIG`, $w^A_{t_s}$ is increased by one. On the other hand, since `GATCCGGCGAA` is in `REPCTX`, the occurrence of t_s in r_2 is not considered in $w^A_{t_s}$, thus avoiding to erroneously overestimate the frequency of allele a_1 of v_2.

We note that on one hand this approach allows us to avoid overestimating the frequencies of some alternate allele but, on the other hand, it produces two major side effects. The first one is that some allele might be underestimated by `MALVA`; indeed, if the k_c-mer covering an alternate allele in a donor is equal to a k_c-mer in the genome it will not be detected. The second side effect is that `MALVA` might overestimate the frequency of some allele due to identical signature. Indeed, suppose that the signature of some alternate allele a_i of another variant $v_j \neq v_2$ is equal to the signature of alternate allele a_1 of variant

v_2. It is obvious that the weights of the k_s-mers of the two signatures will be identical and that the occurrences of both the alleles will concur towards their final value, overestimating it.

Although the two side effects pose some limit to the method proposed in this paper, they arise rarely and we think they are a fair price to pay to avoid biases introduced by the reference genome.

S1.4. Implementation details.

`MALVA` is implemented in `C++` and it is freely available at `https://github.com/AlgoLab/malva`. Bloom filters were implemented as the union of a bitvector and a single hash function `H`. Although it is not conventional, in most cases to use a single hash function has similar results as using multiple ones, as noticed by other authors (Sun et al., 2017; Sun and Medvedev, 2018). To check this claim, while developing the tool we tested whether using multiple hash functions would improve the results by extending the Bloom filters to count-min sketches (Cormode and Muthukrishnan, 2005). As expected, the deterioration of the performance far outweighted the gain in precision and recall (that was less than 0.1%). Moreover, to use a single hash function allows us to store w_t^R and w_t^A efficiently for each k-mer t. Indeed, note that once all the signatures of all the alternate alleles have been added to `ALTSIG`, the latter is only used to check whether some k_s-mer is part of a signature, *i.e.*, it becomes static. By representing `ALTSIG` as a Bloom filter $B_{\texttt{ALTSIG}}$ we can create an integer array `CNTS` of size $\texttt{rank}_1(|B_{\texttt{ALTSIG}}|+1, B_{\texttt{ALTSIG}})$ to store the weights of each k-mer compactly and, if a k-mer t of a signature s is in `ALTSIG` (*i.e.*, if $B_{\texttt{ALTSIG}}[\texttt{H}(s)] = 1$) we can access its weight by accessing $\texttt{CNTS}[\texttt{rank}_1(\texttt{H}(s), B_{\texttt{ALTSIG}})]$. In a nutshell, after adding all the alternate alleles to $B_{\texttt{ALTSIG}}$, we *freeze* it, build a rank data structure over it, compute the number of ones, and create the `CNTS` array of the correct size. Similarly, we implemented `REPCTX` as a Bloom filter $B_{\texttt{REPCTX}}$ using a single hash function. Conversely, `REFSIG` was implemented as a simple hash table, because the number of elements it stores is usually smaller than the number of elements stored in `ALTSIG`. The bitvectors, the rank data structure,

and the CNTS array were implemented using the sdsl-lite library (Gog et al., 2014). We pose an upper limit of 255 to the value of each cell of the CNTS array, so as to store each counter using only 8 bits.

Finally, instead of scanning all the k_c-mers in the read sample, we used KMC3 (Dugosz et al., 2017) to efficiently extract them and counting their occurrences. Therefore, in step 3 MALVA parses the output of KMC3 and updates the counts for each k_s-mer accordingly.

S1.5. Data and Software Availability

MALVA is freely available at https://github.com/AlgoLab/malva. Information and instruction on how to replicate the performed experiments are available at https://github.com/AlgoLab/malva_experiments.

Supplemental References

Bloom, B.H., 1970. Space/time trade-offs in hash coding with allowable errors. Commun. ACM 13, 422–426.

Chikhi, R., Rizk, G., 2013. Space-efficient and exact de bruijn graph representation based on a bloom filter. Algorithms for Molecular Biology 8, 22.

Consortium, .G.P., et al., 2015. A global reference for human genetic variation. Nature 526, 68.

Consortium, I.H., et al., 2003. The international hapmap project. Nature 426, 789.

consortium, U., et al., 2015. The uk10k project identifies rare variants in health and disease. Nature 526, 82.

Cormode, G., Muthukrishnan, S., 2005. An improved data stream summary: the count-min sketch and its applications. J. Algorithms 55, 58–75.

Danecek, P., Auton, A., Abecasis, G., Albers, C.A., Banks, E., DePristo, M.A., Handsaker, R.E., Lunter, G., Marth, G.T., Sherry, S.T., McVean, G., Durbin,

R., Group, .G.P.A., 2011. The variant call format and VCFtools. Bioinformatics 27, 2156–2158.

Dugosz, M., Kokot, M., Deorowicz, S., 2017. KMC 3: counting and manipulating k-mer statistics. Bioinformatics 33, 2759–2761.

Gog, S., Beller, T., Moffat, A., Petri, M., 2014. From theory to practice: Plug and play with succinct data structures, in: 13th International Symposium on Experimental Algorithms, (SEA 2014), pp. 326–337.

Li, H., 2011. A statistical framework for SNP calling, mutation discovery, association mapping and population genetical parameter estimation from sequencing data. Bioinformatics 27, 2987–2993.

McKenna, A., Hanna, M., Banks, E., Sivachenko, A., Cibulskis, K., Kernytsky, A., Garimella, K., Altshuler, D., Gabriel, S., Daly, M., et al., 2010. The genome analysis toolkit: a mapreduce framework for analyzing next-generation dna sequencing data. Genome Research 20, 1297–1303.

Melsted, P., Pritchard, J.K., 2011. Efficient counting of k-mers in DNA sequences using a bloom filter. BMC Bioinformatics 12, 333.

Pajuste, F.D., Kaplinski, L., Möls, M., Puurand, T., Lepamets, M., Remm, M., 2017. FastGT: an alignment-free method for calling common SNVs directly from raw sequencing reads. Scientific Reports 7, 2537.

Shajii, A., Yorukoglu, D., William Yu, Y., Berger, B., 2016. Fast genotyping of known SNPs through approximate k-mer matching. Bioinformatics 32, i538–i544.

Sun, C., Harris, R.S., Chikhi, R., Medvedev, P., 2017. Allsome sequence bloom trees, in: Research in Computational Molecular Biology - 21st Annual International Conference, RECOMB 2017, pp. 272–286.

Sun, C., Medvedev, P., 2018. Toward fast and accurate SNP genotyping from whole genome sequencing data for bedside diagnostics. Bioinformatics 35, 415–420.

Venter, J.C., Adams, M.D., Myers, E.W., Li, P.W., Mural, R.J., Sutton, G.G., Smith, H.O., Yandell, M., Evans, C.A., Holt, R.A., et al., 2001. The sequence of the human genome. science 291, 1304–1351.

Vigna, S., 2008. Broadword implementation of rank/select queries, in: Experimental Algorithms, 7th International Workshop, WEA 2008, pp. 154–168.
